# Observations on the Modus Operandi of Opium

**Published:** 1802-04

**Authors:** M. Ward

**Affiliations:** Manchester


### *Observations on the Modus Operandi of Opium.*

[ Continued from Vol. VII. p. 137. ]

AS I have not repeated the experiments with which I concluded my last paper, I shall only observe, at present, that should they be confirmed by future experiments, we shall be authorized to conclude, that opium, introduced into the system, through the medium of the absorbents, operates *directly* as a sedative.*

I am

---

* I find by the following note which occurs in p. 33, of Dr. Wilson's Essay on Opium, and which I must have overlooked at the time, that Dr. Alexander

I am now to proceed in endeavouring to prove that opium does *not* act as a stimulant.

The 9th Experiment* is liable to the same objections as the 1st, 2d, 3d, and 4th.

Exp. 10.† "Through a very small opening, I injected into the cavity of the abdomen of a rabbit about half an ounce of the same aqueous solution, preventing as much as possible the admission of air. Twenty minutes after the animal was opened, and the surface of the intestines appeared much more red than usual, and apparently inflamed."

So far is this experiment from proving that opium is a stimulant, when *all* the effects which follow its exhibition in this way are considered, that it would have been extremely difficult to have adduced any thing more unfavourable to that opinion.

It has been proved by experiments made on an extensive scale, on different animals, and by some of the first Physiologists of the age, that by injecting a solution of opium into the cavity of the abdomen, the sensibility, irritability, and mobility are *diminished* in a very remarkable degree; effects which cannot be owing to a *stimulant* property in the medicine, as they are not preceded by any symptom of increased action or excitement. The *primary* effect it produces, is a *diminution* in the *frequency and force* of the pulsations of the heart and arteries; the animals soon become *torpid and languid*, and continue in this state as long as life remains.

The following experiment is taken from Dr. Whytt's Essay on Opium,‡ "The same young gentleman, Mr. Ramsay, at my desire, made the following experiment. On the 9th of April, 1755, after making an opening into the cavity of the abdomen of the dog on whom the last experiment was made, he injected by the wound a drachm of opium dissolved in two ounces and a half of water; but before he could stitch up the wound, about an ounce of the solution escaped. The dog lost the *power* of his hinder limbs almost *instantaneously*. Two minutes after the injection was made he began to be convulsed; and

---

Alexander does not assert that opium applied externally is never absorbed, which might be inferred from a remark which occurs in my last paper. Med. and Phys. Journal, Numb. XXXVI, p. 125. "Dr. Alexander was led from his experiments on this head, to conclude upon the whole, that although we cannot positively assert that opium is not absorbed, yet we have no proof of its being so, at least to such a degree as to kill." I am sorry I did not find out the mistake in time to correct it.

* Inquiry, p. 54.

† Inquiry, p 54, 55.

‡ Ess. & Obs. Phys. & Lit. Vol. II. p. 317—329.

and in two minutes more, after having raifed himfelf upon his fore-legs, he *fell down* fenfelefs. At this time Mr. R. laid bare the thorax, by diffecting off the teguments, *which did not seem to give the dog any pain*; and could plainly feel the motion of his heart through the pleura; it beat 76 times in a minute, but became gradually flower.*

"Immediately after counting the pulfe, Mr. R. cut the ribs on each fide of the fternum, which he laid back in the ufual way. The heart, which was thus brought in view, appeared quite turgid, and continued in motion about five minutes; during which time it performed only between 60 and 65 *weak* vibrations; for they were not compleat contractions. While the heart was thus moving, warm faliva was firft applied to it, then cold water, and laft of all oil of vitriol; which fhrivelled the parts it touched almoft in the fame manner as a hot iron would have done; but none of them accelerated the heart's vibrations, *which became gradually slower, till they ceased altogether*. The fibres of fome of the intercoftal mufcles on the right fide of the fternum, continued to be agitated with a weak tremulous motion near half an hour after the injection was made into the *abdomen*; but the intercoftal mufcles attached to the ribs, on the fides of the thorax, were not obferved to move, nor did the diaphragm make any motion when its fibres were pricked or cut.

"*Nothing remarkable was seen in the abdomen*; only, although it was opened ten minutes after making the injection, the *intestines had no motion*; *whereas, in another young dog, which had got no opium, Mr. Ramsay observed the peristaltic motion continue half an hour after laying open the thorax*.

"The dog loft little or no blood in making the wound into his abdomen, nor were any of his bowels hurt by it."

The experiments I fhall next introduce are from Dr. Monro's Effay on Opium.† They are, if poffible, ftill more decifive in proving the *directly* fedative properties of opium.

"To determine this queftion fatisfactorily, it was neceffary to difcover fome other way in which the animal could much more quickly be affected to a violent degree. This I found might be done by pouring thirty drops of the folution of opium, through a fmall hole, into the cavity of the abdomen.‡

(SEVEN

* The dog's heart, in a natural ftate, and before the injection of the folution of opium, beat 150 in the minute.

† Eff. & Obf. Ph. & Lit. Vol. III. p. 317, 318.

‡ "Like to what Drs. Langrifh and Whytt had obferved of lauro cerafus and opium on dogs."

( SEVEN TRIALS. )

" For in *two* minutes thereafter *the heart did not beat above half its usual number of times in a minute, and after four or five minutes, not above a third part of the usual number*; and, on examining the foot with the microfcope, I found that the blood in its veffels HAD ENTIRELY CEASED FROM MOTION. And now the whole mufcles were repeatedly convulfed, and the limbs extended, fo as to render the animal *unable to move out of the place where it lay.**

After a quarter of an hour it fcarcely difcovered any outward fign of life, though the heart was found to contract *feebly* about *ten times in a minute five or six hours thereafter.*"

Dr. Wilfon has been at confiderable pains to afcertain in what manner opium acts in producing the abovementioned phenomena, and has happily fucceeded.†

I fhall only make fuch extracts from his Work, as are neceffary to confirm and elucidate the experiments I have juft cited.

" I therefore repeated Dr. Monro's experiment more than once, but found the refult as he has ftated it. *The beating of the heart became less frequent,* ALMOST IMMEDIATELY, *on injecting the solution into the cavity of the abdomen.*" P. 58.

" Thefe circumftances led me to confider, whether or not there is any other way in which a folution of opium, injected into the cavity of the abdomen, can be fuppofed to influence the motion of the heart, befides through the medium of the nervous fyftem, or that of the abforbents.

" A conjecture occurred to me, which is confirmed by feveral of the following experiments, namely, that opium applied to the coats of the blood veffels, by deftroying their mufcular power, *(opium, it was found, destroys the power of action in all muscles to which it is immediately applied)* muft affect the circulation in thefe veffels; and, confequently, thrown into the cavity of the abdomen, influence the motion of the heart by impeding or entirely interrupting that of the blood, in nearly one third of the whole animal; by which the fupply to the heart is diminifhed, and a greater than ufual obftacle oppofed to its perfect evacuation.

" The firft circumftance, then, to be afcertained, is, whether opium applied immediately, or nearly fo, to the blood veffels of a living animal, impedes or wholly interrupts the circulation

* Frogs were the fubjects of thefe experiments.

† Effay on Opium, p. 57—69.

lation in thofe veffels, independent of any general affection of the fyftem?

"It is to be obferved, that in the following experiment, the opium is not applied immediately to the coats of the blood-veffels, but injected into a cavity, between which and thefe veffels a denfe membrane of cellular fubftance is interpofed. The fkin of a frog, except in a few places, (chiefly the joints) does not adhere to the parts which lie beneath it.

"Having adapted the web of a frog's foot to a microfcope, I injected eight or ten drops of a folution (nearly as ftrong as the ftronger folution) under the fkin of the leg.

"*In a few seconds the circulation became languid, and no motion could be perceived in some of the larger blood-vessels. It gradually became more obscure in the rest, till, in the space of about two or three minutes after the injection of the opium, it ceased altogether.* Nor did this interruption of the circulation proceed from any general affection of the fyftem, fince the motion of the blood ftill continued in the other foot.

"Left it might be fufpected, that in applying the foot to the inftrument the veffels were compreffed, it is proper to obferve, that the circulation in the foot, after it was applied to the microfcope, continued as vigorous as natural, till the folution was injected. On another occafion, indeed, I have obferved the circulation in the foot of a frog, applied in the fame way to the fame microfcope, vigorous for feveral hours.

"This experiment was made three times, in the fame manner, and with the fame refult.

"After determining that opium is capable of interrupting the circulation in the part to which it is immediately applied, independent of any general affection of the fyftem; all that is neceffary, in order to afcertain whether it is in this way that it fuddenly affects the motion of the heart, when thrown into the cavity of the abdomen, is to interrupt the circulation, and obferve the effects of this drug, applied in the fame way, when it can only act on diftant parts, through the medium of the nervous fyftem." P. 59—63.

A particular account of the experiments immediately fucceeds (p. 64—68.) The following is the conclufion they fuggeft.

"The inference from thefe experiments is, that the *diminished frequency of the heart's motion, observed almost immediately on throwing a solution of opium into the cavity of the abdomen, does not proceed from any action of the opium on this organ, through the medium of the nervous system, but from its impeding or entirely interrupting the circulation in nearly one-third of the whole animal.*" P. 68, 69.

Effects nearly fimilar were obferved to refult from injecting opium into the abdominal cavity of guinea-pigs, by the Abbé Fontana.

His words are, "Thofe into the bellies of which the aqueous folution of opium was injected, died in lefs than two hours: *They lost the greater part of their motion in less than half an hour*, and were violently convulfed."*

On fumming up the evidence derived from the above experiments, it appears that opium injected into the cavity of the abdomen of different animals *deprives them of voluntary motion almost instantaneously*; *that it impedes or interrupts the circulation of the blood, in the vessels of the abdominal viscera*; *the pulsations of the heart almost immediately become feeble and slow, and the circulation of the blood ceases in the extremities*; *the peristaltic motion of the intestines is retarded and destroyed: The sensibility and irritability are impaired to such a degree, that the dog*, in Mr. Ramfay's experiments, *did not seem to feel any pain when that gentleman laid bare the thorax, by dissecting off the teguments, though the heart was beating* 76 *times in a minute at the time*; *nor did the diaphragm make any motion when its fibres were pricked or cut.*

Dr. Crumpe's view in making the experiment feems evidently to have been, to obferve the *local appearances* refulting from the application of a folution of opium to a tender irritable furface; and this accounts for his omitting to notice any other circumftance. But after having *proved to a demonstration*, that the *primary and general* operation of opium injected into the cavity of the abdomen is DIRECTLY AND POWERFULLY SEDATIVE (both the voluntary and involuntary motions yielding to its influence) it neceffarily follows, that the *increased redness* and *apparent inflammation* † could not have been the confequence of the opium having acted as a ftimulant, unlefs we fuppofe that it acts as a ftimulant and a fedative *at the same time*, which would be abfurd; and, indeed, Dr. Crumpe has fhewn that idea to be unfounded.‡

As

* Treatife on Poifons, Vol. ii. p. 363. (Skinner's tranflation.)

† Thefe appearances feem not to have occurred in Mr. Ramfay's experiment. Dr. Whytt fays, nothing remarkable was feen in the abdomen; only, although it was opened ten minutes (20 minutes had elapfed in Dr. Crumpe's experiment) after making the injection, the inteftines had no motion.

‡ "It is in the firft place to be remarked, that opium cannot be feparated into any two principles, endowed folely with the oppofite qualities of a ftimulant and fedative, as will be feen by recurring to the chapter in which

As they cannot be explained therefore by fuppofing the modus operandi of opium to be ftimulant, it may be worth while to enquire, whether they do not admit of a fatisfactory explanation, by fuppofing it to act as a fedative.

We have feen, *that opium destroys the muscular power of the coats of the blood vessels*, though it is *not* applied *immediately* to their coats, but injected into a cavity, between which and thefe veffels, a denfe membrane of cellular fubftance is interpofed.*

It therefore follows, from the unequivocal manner in which this medicine acts, that this effect will be produced in a greater degree, when the folution is applied *immediately*, or nearly fo, to their coats, as it is to thofe of the peritoneum when injected into the cavity of the abdomen; confequently, the veffels having loft their *tone* as well as their *action*, they will appear enlarged, and more red than ufual: Veffels which were before invifible to the naked eye, will now be readily diftinguifhed, and the parts may thus affume an inflamed appearance, fimilar to what takes place when an aqueous folution of opium is applied to the tunica fclerotica of a found eye, or to the cutis, recently deprived of the cuticula.

The above explanation was fuggefted by the experiments which have been adduced to prove, that opium injected into the cavity of the abdomen does *not* act as a ftimulant: It has one advantage, (a negative one to be fure) *it is not at variance with established facts*; but however unfatisfactory it may be deemed, that will not tend to invalidate either the experiments or the arguments which have been employed.

---

which its analyfis is contained. The principal argument on which thofe who attribute to this medicine both ftimulant and fedative qualities rely, is deduced from its being found firft to increafe, and afterwards diminifh the frequency of the pulfe: But, as will be hereafter fhown, this effect can be explained in a much more fatisfactory manner, fo that there is no neceffity of reforting in this cafe to a fuppofition, rendered highly improbable from confidering, that we know of no fimple in the whole Materia Medica poffeffed of powers in their nature oppofite, and operating, when it is exhibited, feparately and diftinctly. But even granting that opium is endowed with thefe contrary qualities, it will be difficult to explain on fuch data the confequences of its exhibition; for as the effects of fuch principles are fuppofed to be diametrically oppofite, it naturally follows that one muft counteract the other; and hence, if both exift in equal proportions, the medicine would turn out perfectly inert; if one preponderates, it will act folely in confequence of the excefs of that principle; and to it alone, whether ftimulant or fedative, will all its effects be afcribable." Inquiry, p. 167, 168.

* Wilfon's Effay on Opium, p. 60—63.

EXP. II.* "I raifed the fternum and laid bare the contents of the thorax of a dog which had been juft hanged; the heart was ftill contracting, but in a very irregular manner. I opened the pericardium and waited till all contraction ceafed, and then let fall on it a few drops of the aqueous folution, milk warm. The contractions were inftantly renewed and continued for about a minute, and the furface of the heart became unufually red. Upon the contractions ceafing, they were again renewed, but for a fhort time, by a fimilar application: A third attempt was ineffectual; but on injecting a fmall quantity into the fuperior venous finus, the whole heart was again thrown into action, which continued for a fhort time, and was the laft effort it could be roufed to. I made a fimilar experiment with plain warm water. Its firft application produced a flight renewal of the heart's contractions, the fecond and third were ineffectual."

Here fome other caufe muft have been applied, that alleged being inadequate to the effects produced.

"I opened the pericardium (fays Dr. Crumpe) and waited till all contraction ceafed, and then *let fall* on it *a few drops* of the aqueous folution, milk warm. The contractions were *instantly* renewed, &c."

Now it is a well-known fact, that the fhock occafioned by allowing any liquid to *fall* upon a part poffeffing fenfibility is a powerful *stimulus*; befides, is it probable that a *few drops* of a folution of opium, containing *five-eighths of a grain to ten drops of water*, † could produce *any sensible effect*, applied to the external furface of the heart of a dog, in the fituation here defcribed, (whatever may be the modus operandi) *if applied without the addition of mechanical force?*

"Upon the contractions ceafing, they were again renewed, but for a fhort time, by a fimilar application: A third attempt was ineffectual; *but on injecting a small quantity into the superior venous sinus, the whole heart was again thrown into action*, which continued for a fhort time, and was the laft effort it could be *roused* to."

I fhall be able to adduce the moft pofitive and unequivocal proofs, that an aqueous folution of opium applied to the internal furface of the hearts of frogs, *(no mechanical force being superadded)* INSTANTLY *puts a stop to their contractions.*

Dr.

* Inquiry, p. 55, 56.

† "This folution, and that ufed in all the fucceeding experiments, was made by diffolving opium in warm water, in the proportion of one drachm of the former to two ounces of the latter." Inquiry, p. 24.

Dr. Crumpe adds, "I made a fimilar experiment with plain warm water. *Its first application produced a slight renewal of the heart's contractions*; the fecond and third were ineffectual."

And the inference he draws from this experiment is, that "*Dropped* on the hearts of animals, *it accelerates their motions or rouses them into action if they have previously ceased to vibrate.*" P. 169.

And yet the only inference that can be drawn from the 30th experiment, (p. 129, 130) and which Dr. Crumpe evidently intends his readers to draw from it, is, that opium *diminishes the irritability and power of motion* in the hearts of frogs, when applied to them immediately after their feparation from the body. But I fhall give the experiment and his arguments, verbatim.

Fontana concludes from experiments related in his Supplement, that, "The hearts of animals taken from the body, and confequently deprived of blood, when immerfed in watery folutions of opium, are *not* deprived of *irritability* and *the power* of *motion*, fooner than hearts immerfed in pure water."*

"This fact (fays Dr. Crumpe) I am under the neceffity of denying, although the learned Haller is of the opinion, as well as the Abbé Fontana. Whytt afferts, from the refult of feven experiments which he particularly details, that the *irritability and motive faculty* of the heart is, *as well as that of other muscles*, DESTROYED by opium.† In determining this point, it is to be obferved, that if the hearts of a number of frogs, the animals on which fuch experiments are ufually made, be feparated from the body, though no application whatever be made to any of them, fome will die, or lofe their irritability, many minutes before others. It is therefore only from many experiments, and from taking a general view of the whole, that we can determine the point in queftion; and after having tried the experiment upon a great number of hearts, I do not hefitate to declare, that the *irritability* of that organ is *destroyed* by opium, *long before it would naturally disappear.* I think I have in general obferved, that the larger the animal, the longer does its heart, when feparated from the body, continue to vibrate; and in comparing the effects of plain water and opium, we

* It has been fully afcertained by experiments made both before and fince, that this conclufion of Fontana's is erroneous; but this very circumftance (as muft by this time appear, and will be fhewn more fully afterwards) throws another infurmountable difficulty into the way of Doctor Crumpe's Theory.

† "Effays Ph. & Lit. V. ii. Art. xx."

we ſhould, as nearly as poſſible, match the hearts employed in ſize. The liquors ſhould alſo be of the ſame temperatures, as a variation in this point will occaſion a manifeſt difference.

"With theſe precautions in view were the experiments above-mentioned conducted. The following are the particular reſults of the laſt inſtituted on the ſubject."

Exp. 30. "Having taken three frogs, one of a very large, another of the middle, and the third of rather a ſmall ſize, I ſeparated the hearts from their bodies, and immerſed them in pure water. The firſt lived *forty-five* minutes; the ſecond and third *forty* minutes. Immerſing the hearts of three frogs, of nearly equal ſizes, in a watery ſolution of opium; the firſt lived but *twenty* minutes, the ſecond *eleven*, the third ſcarcely *ten*." Inquiry, p. 127, 130.

After what has been advanced, it muſt appear ſufficiently evident, that on the preſent occaſion there is a want of conſiſtency in Dr. Crumpe's arguments; and the following experiments ſhew, in a clear and deciſive manner, how far his 11th experiment is capable either of elucidating the ſubject of the modus operandi of opium, or of ſupporting the theory for which Dr. C. contends.

"It remains to examine (ſays Dr. Monro) how far animals are affected by the abſorption of opium, and its mixture with their circulated blood.

"But previous to this inquiry, I thought it might be of uſe to know what effect opium has when applied to the heart and vaſcular ſyſtem.

( Four Trials. )

I therefore laid bare the heart, and then injected into a large vein, which runs along the under middle part of the abdomen on the outer ſide of the peritoneum, a few drops of the ſolution,* viz. twelve drops into that vein of two frogs, and ſix into that of two others; and obſerved, that *as soon as the solution had entered the ventricle of the heart, that organ was rendered incapable of expelling its contents*; *and*, *in much less than one minute thereafter*, *became so entirely paralytic as not to make the least contraction on the strongest irritation*, *whether applied to its outer or inner part*; whilſt, at the ſame time, the extremities were agitated inceſſantly with convulſions, which continued for about three-quarters of an hour, gradually becoming more and more feeble; *nor was the animal able to perform any voluntary motion during this whole period.*

"Remarks.

* Conſiſting of a drachm of opium to an ounce of water.

"Remarks.

"1. When we compare that part of the foregoing experiment which relates to the heart, with ſome experiments made by Dr. Whytt, when the opium was applied to its outer part, we ſee how greatly the delicacy of feeling of the inner ſide of the heart exceeds that of its outer ſide.

"2. We learn that the heart is ſo far from being exempted from the influence of opium, as the learned Dr. Haller ſuppoſes, that, perhaps, *no muscle of the body can by any means be proved to be so much under its dominion as the heart, when the opium is brought into contact with its inner side*; in which way it will be applied if abſorbed."*

The following experiments are equally applicable to the preſent purpoſe.

"After ſecuring the aorta by ligature in twelve frogs of different ſizes, a few drops of the ſtrong ſolution of opium was injected into the heart of each. *It immediately ceased moving*. This, however, was not followed by the ſlighteſt convulſion in any part of the body.

"The frogs all died in exactly the ſame manner as theſe animals do when the heart is cut out; that is, when the circulation is interrupted, and when they loſe as much blood as in this experiment.

"I could not perceive that the injection of the ſolution had any effect, *but that of putting a stop to the motion of the heart*.

"A ſimilar experiment was made in the following manner: After dividing the aorta in ten frogs, moſt of them full grown, a few drops of a ſolution of opium, (not quite ſo ſtrong as that uſed in the laſt experiment) were thrown into the heart of each. Its contractions instantly ceased, but no convulſions ſupervened."

I ſhall paſs over the remarks which immediately ſucceed the above experiments.

"This, however, ſuggeſted another mode of making the experiment, which ſeems perfectly concluſive.†

"I ſlit the heart in ſix frogs. Notwithſtanding its contents were thus inſtantly evacuated, *it continued to contract with vigour*. A little of the ſame ſolution uſed in the laſt experiment was then dropped into it.

"No

---

* Eſſays, Ph. & Lit. Vol. iii. p. 330—332.

† Theſe experiments were made with a view to aſcertain how far opium, applied to the heart, is capable of affecting any diſtant part through the medium of the nervous ſyſtem.

" No part of the ſolution, applied in this way, can be ſent through the arteries to the brain; but as all the nerves are left entire and uncompreſſed, if the convulſions which follow the injection of opium into the heart depend on any action of that drug on the nervous ſyſtem, they ought to be obſerved in this experiment.

" On dropping the ſolution into the heart, *it immediately ceaſed moving*, but no convulſions ſupervened."†

To attempt any comments upon the above important and deciſive experiments, would only weaken their force; I ſhall, therefore, only add, that it is *fully aſcertained by thirty-two experiments, that opium, applied to the hearts of animals,* IMMEDIATELY PUTS A STOP TO THEIR MOTIONS; *and it is alſo aſcertained, on the authority of four experiments, that they become ſo entirely paralytic in leſs than one minute thereafter, as not to make the leaſt contraction on the ſtrongeſt irritation, whether applied to their inner or outer part.*

" It has been ſhewn (ſays Dr. Wilſon) that opium, applied *to the external ſurface of the heart, very little, if at all, affects the muſcular power of this organ*; applied in conſiderable quantity to its *internal ſurface, it immediately deſtroys it.*"‡

Dr. Whytt's experiments upon the hearts of frogs, *ſeparated from the body*, and immerſed in a ſolution of opium, ſhew very clearly that opium directly *diminiſhes* their mobility, ſenſibility, and irritability; but the fact is ſo generally known and acknowledged, that I ſhall paſs on to his concluſion, p. 335.

" It appears, beyond doubt, from the preceding experiments, that the heart is not exempted from the power of opium, as the learned Dr. Haller has affirmed,§ but has *its motion deſtroyed by it, as well as the other muſcles, only not ſo ſoon.*"

EXP. 12. ‖ " Having ſeparated, by inflation, the ſkin and muſcles of the lower extremities of a frog, I poured between them a quantity of the watery ſolution; both limbs were deprived of the power of motion in about nine minutes; the animal died in fifty-five minutes."

The plain and neceſſary inference ſuggeſted by this experiment, is diametrically oppoſite to the concluſion Dr. Crumpe endeavours to eſtabliſh.

It is evidently his deſign to prove, that opium is a *ſtimulant*; and, as a proof of the *validity* of the doctrine, we find, that on pouring

† An Eſſay on Opium by A. P. Wilson, M. D. p. 48—50, and 53, 54.

‡ Eſſay, p. 85.

§ Act. Gotting, Vol. ii, p. 147 and 154.

‖ Inquiry p. 56.

pouring a folution of opium between the fkin and mufcles of the lower extremities of a frog, *both limbs were deprived of the power of motion in about nine minutes.*

Thefe, to be fure, are *strong proofs of a* STIMULANT OPERATION ; and when feveral *other consequences* of injecting opium between the fkin and mufcles of frogs (which occurred to Dr. Monro) come to be added, without doubt they muft appear in the aggregate perfectly convincing.

In the following experiments the effects of the opium were not confined to the limbs, but acted generally.

" Exp. 6. I next endeavoured to afcertain the effect of the opium on the mufcles when immediately applied to, or in contact with them, and how far the nerves of the fkin would fuffer by applying the opium to its inner fide.

" For this purpofe, I cut a hole in the fkin at the top of the thigh, and poured the opium into the cellular membrane, between the fkin and mufcles.

(Three Trials.)

" I firft poured forty drops of the folution under the fkin of the left thigh and leg, having before hand broke, with a probe, a partition between thefe made by the cellular fubftance of the knee.

" I did not attend to the effects till half an hour thereafter, when I found that whole member paralytic, its toes and fkin having *lost their sensibility*, and the mufcles their motion. *The animal seemed much stupified*, and could fcarcely move its body from the place by the help of the other hind extremity; *and the blood had ceased from motion in the small vessels of both feet; though on examining the heart, I found it still gave twenty-two regular, but feeble, strokes in the minute.* After ten minutes more, all the members of the body were extended and convulfed, and the animal was quite unable to move its body out of the place where it was laid, and foon after died."

(Two Trials.)

" I then tried the fame experiment with twenty drops only of the folution, and obferved, that *in about a quarter of an hour that extremity was much weakened and less sensible*, and in five minutes more was not only *motionless and insensible, but the animal seemed to be much stupified and lay still*, unlefs when it was hurt, and after nearly the fame time as thofe above, had its members extended and convulfed, and foon expired.

(Two Trials.)

After that I tried the fame experiment with *ten* drops only. After twenty minutes, *that leg seemed to be weaker, and in ten minutes more, its muscles had lost their power, and the toes had*

*little sensibility*; *and now the animal seemed to be a good deal stupified, and the heart of one of them examined gave only* 25 *strokes in a minute.* An hour and a half after the beginning of the experiment, the toes feemed to have *quite lost their sensibility, and the muscles their motion*; *but the other parts were not convulsed,* and the animal jumped by the help of the other hind extremity. *And two days thereafter, this leg had recovered both its sense and motion,* and the animal feemed quite well, and continued fo for fourteen days at leaft.

(Two Trials.)

"In the laft place, I poured ten drops under the fkin of the thigh only in one frog, and the like quantity under the fkin of the leg only in another, without finding that either of thefe members loft their fenfe or motion, or that the reft of the body was obfervably affected."*

In the following experiment the modus operandi is very clearly illuftrated. In this inftance the effects of the opium are confined to the limb.

"Having adapted the web of a frog's foot to a microfcope, I injected *eight or ten drops* of a folution, (nearly as ftrong as the ftronger folution) under the fkin of the leg.

"In a few seconds *the circulation became languid, and no motion could be perceived in some of the larger blood vessels. It* gradually *became more obscure in the rest, till, in the space of about two or three minutes after the injection of the opium,* it ceased altogether. Nor did this interruption of the circulation proceed from any general affection of the fyftem, fince the motion of the blood ftill continued in the other foot.

"This experiment was made three times in the fame manner, and with the fame refult."†

It muft appear extraordinary after perufing the 12th, and efpecially when we come to confider the 30th, 32d, 36th, and 42d of Dr. Crumpe's experiments, that he fhould have expreffed himfelf in fuch *unqualified terms,* as he every where does, when fpeaking of the modus operandi of opium.

P. 168, he fays, "The facts and arguments I am about to relate have induced me to conclude, that all its effects are the refult of a *stimulant property alone.*" And yet, Dr. Crumpe is fo far from denying the exiftence of fedatives, that he allows the confequences refulting from fourteen of his own experiments, to denote a *directly sedative operation*; as will be fhewn

* Effays Ph. & Lit. v. 3, p. 309—312.

† Wilfon's Effay on Opium, p. 61—63.

ſhewn when we come to conſider the 32d and the ſucceeding experiments. The reaſon will alſo then appear, which induced him to draw a concluſion that is *contradicted even by his own experiments*, and ſtill more pointedly *by a large body of indubitable evidence*, collected from various, and from the moſt reſpectable, ſources of intelligence.

Exp. 15.* "The pure reſin of Exp. 13, when rubbed between the fingers, had more of the peculiar ſmell of opium than the gum; when chewed, it had no peculiar bitterneſs or pungency. The gum of Exp. 14, had more the ſmell of extract of liquorice than opium; to the taſte it was extremely bitter and pungent."

I have introduced this Experiment for the purpoſe of remarking, that though the activity of opium reſides chiefly, if not entirely, in the reſinous part, as is proved by the 17th and 18th of Dr. Crumpe's Experiments; yet that it poſſeſſes no peculiar bitterneſs or *pungency*; and that the gum, when ſeparated from the reſin, though it is inert, or nearly ſo, compared with the reſin, yet to the taſte it is *extremely* bitter and *pungent*.

Exp. 17.† "Of the pure reſin, procured by the means mentioned in Exp. 13, I took two grains diſſolved in a very ſmall quantity of ſpirit of wine, at one in the afternoon, my pulſe beating 70 in a minute:

| In | 5 | 10 | 15 | 20 | 25 | 30 | 35 | 40 | 45 | 50 | 55 | 60 | 75 | 90 | Min. |
|---|---|---|---|---|---|---|---|---|---|---|---|---|---|---|---|
| P. beat | 78 | 78 | 80 | 78 | 78 | 76 | 70 | 72 | 66 | 65 | 63 | 65 | 63 | 66 | |

"In fifteen minutes, there was an evident increaſe in the fulneſs as well as frequency of my pulſe; in 60, perceived a pleaſing languor and drowſineſs; in 90, this was ſo much increaſed, that I lay on the bed and ſlept for an hour; on getting up found myſelf languid, very thirſty, and affected with a ſlight vertigo and lightneſs of my head; on taking a glaſs of Port wine all theſe ſymptoms were relieved, and I ate my dinner with as good an appetite as uſual."

The only mark of a ſtimulant operation, either in this, or in any of Dr. Crumpe's experiments, (or indeed in any experiment I have met with on the ſubject) which has an *unequivocal* claim to be conſidered as ſuch, is a ſlight and tranſitory increaſe in the frequency, ſtrength, and fulneſs of the pulſe. Now, did this effect *constantly* follow the internal uſe of opium, which is well known to be by no means the caſe, of what force or conſequence could it be of, in whatever point of view it might be conſidered, when contraſted with the many *clear and*

* Inq. p. 62. † Idem. p. 65.

 *decisive*

*decisive marks of a* SEDATIVE *operation, which* INVARIABLY *follow, whenever opium is taken in a sufficient dose to affect the system at large?*

Befides, it will be demonftrated (if it has not been done already) that in every *other* practicable mode of introducing it into the fyftem of different animals, (except by injecting it into the fkull, or into the fpine) whether it be injected into the cavity of the abdomen; into the blood veffels; between the fkin and mufcles; into the rectum; applied immediately to the internal furface of the heart; or externally to the fkin, *it* DIRECTLY DIMINISHES *the frequency and force of the heart's contractions, and produces immobility, and total insensibility* to ftimuli of every kind; and thefe effects *(diametrically opposite to those produced by stimulants)* commence in fome of thefe experiments in a few feconds, and in ALL of them fpeedily, after the introduction of the opium, and increafe gradually or rapidly, and are temporary or permanent, according to the quantity of opium which has been introduced, or the ability of the animal to fuftain its power.* And the introduction of the practice of applying opium externally by means of friction, has furnifhed us with a very ftrong argument againft the ftimulant theory, for in this mode of applying it, it is conveyed by the lymphatics to the thoracic duct, and being there mixed with the chyle and lymph, paffes with them into the fubclavian vein, and from thence to the heart, the brain, and every part of the body.

Thus, as foon as it quits the abforbent fyftem of veffels, (where I apprehend it undergoes no material change, except dilution) it is almoft immediately applied to the centre of the fanguiferous and nervous fyftem; confequently, its genuine effects, whether ftimulant or fedative, muft infallibly appear; and there can be no difficulty in deciding whether it *increases* the mobility, and *excites* the motion of the nervous power; or *diminishes* the fenfibility and irritability of the fyftem, and *thereby the motions and powers of motion in it.*

A brief recapitulation of the refult of two Experiments I lately made,† will place the matter in fo confpicuous a light, as to prevent even the poffibility of a miftake.

The

---

* Opium injected into the fubftance of the brain, or through the fpine, in frogs, neither retards nor accelerates the action of the heart; the reafon of which is obvious; in this way of applying it, it can only act upon the heart through the medium of the nervous fyftem. See Wilfon's Effay on Opium, p. 74—85.

† Medical and Phyfical Journal, Vol. VII, p. 136, 137; and Vol. VI, p. 478—486.

The firſt was made at 6 P. M.—Before the commencement of the friction, the pulſe was 108, firm and regular. In ten minutes, when nearly half of the tinct. of opium was rubbed in, it was reduced to 88, and was ſoft. In twenty minutes it was reduced to 84, was ſtill ſoft, and Mr. Hall (the ſubject of the experiment) was affected with drowſineſs, complained of being cold, and his eyes had a dull heavy aſpect (a common occurrence when opium is introduced by friction). In thirty minutes, he complained of being cold and drowſy; pulſe 84, and ſoft. In forty minutes 84, but weaker; ſtill cold and drowſy. In fifty minutes, pulſe 72, feeble and ſmall; countenance pale; coldneſs continues, and is accompanied with diſagreeable ſenſations. In ſixty minutes, p. 76, weak, and not quite regular; complains of great debility. In ſeventy minutes, p. 90, regular, and rather ſtronger. In ninety minutes, p. 92. In one hundred and twenty minutes, p. 96; ſtill complains of being cold, and the diſagreeable ſenſations did not go off till after ſupper, when his appetite was *better than uſual.*

The ſecond Experiment was made before he had breakfaſted or uſed any exerciſe; the tinct. of opium was increaſed from two to three drachms, and the mixture was made warm before it was applied; the pulſe was 95. In fifteen minutes, p. 86, ſoft, and eaſily compreſſed. In thirty minutes, p. 83, weak and ſoft; he complains of being very weak, cold, and ſhivery; countenance looks ſickly, ſkin feels cold. In forty-five minutes, p. 83, ſoft; complains much of being cold all over, but particularly his feet; is ſhivery and very weak, ſpirits depreſſed, feels drowſy, eyes look heavy. In ſixty minutes, p. 90, weak and ſmall; back painful; complains of being affected with a greater degree, and a different kind, of coldneſs to any he ever experienced before. I now aſked if he had felt exhilarated at all, either in this or the former experiment? He anſwered, No; and could not help ſmiling at the queſtion, as indeed he had reaſon. He ate his breakfaſt with an *uncommon appetite*, and was ſoon relieved from every diſagreeable ſenſation.

The efficacy of opium applied externally, having been demonſtrated in a variety of morbid affections, and its having produced, in every inſtance, a ſeries of conſequences directly the reverſe of ſtimulant, renders the above experiments, in my mind, perfectly concluſive; becauſe, having only to paſs through the lymphatic veſſels in its way to the heart, it is much leſs adulterated than when taken internally; being then expoſed to the action of the gaſtric juice in the ſtomach, and afterwards mixed with the groſſer parts of the food, the bile, &c. It is therefore demonſtrable, that when opium does accelerate the circulation, raiſe the ſpirits, or increaſe the heat of the body, theſe thefe effects muft proceed, either from its immediate and topical action as a fedative upon the primæ viæ, or from fome other *adventitious* circumftance, and not from its operating as a ftimulant.

As this is a point of primary importance, and may have fome weight in enabling us to form a juft decifion, I fhall beg leave to fuggeft a few remarks, which may perhaps affift in the inveftigation.

Does not an aqueous folution of opium deftroy the power of action, in all mufcles (not excepting the heart) to which it is immediately applied? *

Does it not produce the fame effect upon the blood-veffels of the lower extremities, when injected between the fkin and mufcles? †

Does it not put a ftop to the periftaltic motion of the inteftines, almoft immediately on being injected into their cavity? ‡

And does it not operate in the fame manner, but in a lefs degree, when injected per anum? §

Is there any effential difference in the ftructure of the ftomach and inteftines? ||

Has not the ftomach a vermicular or periftaltic motion as well as the inteftines?

Can the functions of the one be affected independently of thofe of the other?

If thefe queftions be anfwered in the affirmative, no farther proofs can be required, to convince us, that a folution of opium applied to the internal furface of the ftomach, muft diminifh the fenfibility and irritability, and thereby the motions and powers of motion, not only in that organ, but in the fyftem at large; or in other words, that it muft act in this, as in every other inftance, directly as a fedative.

This theory will be found confiftent with the experiments as well as with the obfervations of different writers; or may rather be confidered as the natural refult of both.

"The

---

* Whytt's Eff. loc. cit. p. 323—325. Monro's Eff. loc. cit. p. 309—314, 330, 331. Wilfon's Eff. p. 48—50, 53, 54, 60, 85.

† Monro's Eff. p. 309, 310. Wilfon's Eff. p. 59—63.

‡ ——, "Applied to the external furface of the inteftines, I could not be certain that it at all diminifhed the periftaltic motion; on injecting it into their cavity, they almoft inftantly became paralytic." And in a note, Dr. W. fays, "From this effect of opium on the internal furface of the inteftines, we are at no lofs to account for the coftivenefs which attends the ufe of this drug." Wilfon's Eff. p. 85, 86.

§ Monro's Effay, p. 314, 315.

|| Except that the inteftines are not furnifhed with a fet of veffels which fecrete a liquor fimilar to the gaftric juice, and this difference is of no importance on the prefent occafion.

" The virtues of opium, (ſays Dr. Alſton) internally taken, *depend chiefly on its action or influence on the stomach.* I have often obſerved a violent teneſmus removed *in a moment* by a few drops of liquid laudanum, vomiting ſtopped, pain eaſed, yea and ſleep procured the ſame way, *and almost as soon.*" †

Every medical practitioner can bear teſtimony to the truth of theſe obſervations.

" In preſence of, and aſſiſted by Mr. Robert Fullarton, a curious gentleman, and very dexterous in microſcopical obſervations, (in Auguſt, 1733,) I conveyed through a ſmall glaſs tube a few drops of a ſolution of opium in water into a frog's ſtomach, and putting the animal into a glaſs cylinder, adapted it ſo to a good microſcope, that we had a diſtinct view of a part of the membrane betwixt the toes of its hinder foot, where the circulation of the blood may eaſily be ſeen. My deſign was, ſince I found opium killed frogs, to obſerve if there was any viſible change made by it in the blood itſelf, or in its motion; neither of us could indeed ſee any alteration of the blood, as to its conſiſtence, colour of the ſerum, magnitude, figure or colour of the red globules; *but we very distinctly saw a surprising diminution of the blood's velocity, for it did not move half so swiftly as it uses to do in these creatures. We alternately looked at it again and again, and in less than half an hour saw the velocity of the blood gradually increase, the uneasy frog recover its wonted vigour, and the blood its common celerity*; upon which we took out the paddock, put it in a baſon of clean water, and allowed it half an hour to refreſh itſelf; *then gave it another dose of opium, fixed it to the microscope* WITH ALL EXPEDITION, *and viewed it as before*; *the blood then moved yet slower than it did the first time, and its velocity gradually decreasing, at length it stagnated, first in the smaller then in the larger vessels*, and in about a quarter of an hour the animal expired. One thing was very obſervable all along, viz. that notwithſtanding the diminiſhed velocity of the blood, there was no ſenſible diminution of the frequency of the pulſe; yea, when there was no circulation or progreſſive motion of the blood in this part, the pulſe was viſible by an undulatory motion; that is, *the blood returned as far back at every diastole of the heart as it was protruded by the preceding systole*; *this continued till the frog was quite dead, or at least appeared to be so.* When we had loſt all hope of its recovery I opened it, and found nothing in its ſtomach but a clear mucus like a jelly, a little coloured with the opium, of which it was full; every thing elſe ſeemed perfectly

† Medical Eſſays, vol. v. part i. p. 165.

perfectly natural. *This experiment we frequently repeated, and it had always the same appearances and event.* The recovery, however, of one of the frogs, which for a confiderable time feemed to be dead, is not to be omitted. My friend and I one evening killed, as above, a couple of frogs with opium; one of them, which was the ftrongeft, I laid half in water on a tile, in the bottom of a water pot, that if it recovered it might fit either wet or dry, as it liked beft; the other I left on the earth dry under a hedge. Next morning, when I returned to the garden, I found the one under the hedge dead as I left it, but the other in the water pot was alive and appeared to be in perfect health.

While we were thus employed, another thing occurred, which though foreign to the prefent fubject, it may not be amifs to mention. One of the frogs we got for the above experiments, *had not the use of one of its hinder legs, which was of a pale reddish colour.* This made me defirous to obferve by the microfcope the circumftances of the circulation in this paralytic, *and apparently inflamed member; and we found that the red globules were entirely dissolved; that the blood-vessels were distended with a reddish homogeneous liquid, as if the part had been injected with a bloody water; and that neither sense nor motion remained in it.*"‡

In the beautiful experiment juft related, though it was frequently repeated and always with the fame appearances and event, we do not find the circulation was accelerated, after the folution was thrown into the ftomach of the frog; but on the contrary, that Dr. Alfton and Mr. F. very diftinctly faw (from the firft) *a surprising diminution of the blood's velocity*; they alternately looked at it again and again, *and in less than half an hour* (when the action of the opium was over,) *saw the velocity of the blood* GRADUALLY INCREASE, THE UNEASY FROG RECOVER ITS WONTED VIGOUR, AND THE BLOOD ITS COMMON CELERITY; and, (which compleats the illuftration) after allowing the frog time to refrefh itfelf, they gave it another dofe of opium, fixed it to the microfcope, *with all expedition,* (five or fix minutes might probably fuffice,) and viewed it as before; *the blood then moved yet slower than it did the first time, and its velocity gradually decreasing, at length it stagnated, first in the smaller, then in the larger vessels, and in about a quarter of an hour,* the animal expired."

*Manchester, March* 9, 1802. M. WARD.

[ To be continued. ]

‡ Medical Effays, vol. v. part i. p. 153—156.

